# Early-onset juvenile nasopharyngeal angiofibroma (JNA): a systematic review

**DOI:** 10.1186/s40463-023-00687-w

**Published:** 2023-12-19

**Authors:** Matthew Newman, Thomas Boi Vu Nguyen, Tobial McHugh, Kesava Reddy, Doron Dov Sommer

**Affiliations:** 1 Otolaryngology-Head and Neck Surgery Division, Department of Surgery, McMaster University Medical Centre, 3V1 Clinic, 1200 Main St. West, Hamilton, ON L8N 3Z5 Canada; 2 Neurological Surgery Division, Department of Surgery, McMaster University, Hamilton, ON Canada

**Keywords:** Juvenile nasopharyngeal angiofibroma, Early-onset, Radkowski stage, Recurrence, Endoscopic, Skull base, Neoplasm, Pediatric

## Abstract

**Background:**

Juvenile Nasopharyngeal Angiofibroma (JNA) is a fibrovascular tumor of the nasopharynx that classically presents in adolescent males. The reported mean age of onset is between 13 and 22 years old [1–6]. Significant androgen stimulation is hypothesized to explain the strong predisposition for JNA to present in young adolescent males. However, considerable variability in age at diagnosis exists with rare involvement of very young patients incongruent with typical male pubertal growth patterns.

**Objective:**

The purpose of this systematic review is to identify cases of early-onset JNA (EOJNA), (defined as age < 10 years) in the literature and to examine the disease characteristics and treatments used in this patient group. A case of a 7 year old boy with EOJNA at our institution is also described and presented.

**Methods:**

We searched Embase, Cochrane database and MEDLINE from 1996 to February 2021 for studies that reported cases of EOJNA. Relevant clinico-demographic data, disease severity and treatment outcomes were recorded and analyzed using descriptive statistics. We compared our findings with reported means for JNA in all ages.

**Results:**

We identified 29 studies containing a total of 34 cases of EOJNA. The vast majority (31/34) of patients were males and the mean age of diagnosis was 8.15 years old. The most common presenting symptoms were nasal obstruction (65.2%) and epistaxis (60.9%). Patients were most commonly Radkowski stage II (39.4%) and III (39.4%). Primary treatment modalities included open surgery (66.7%), endoscopic surgery (24.2%), and radiotherapy (9.1%). Recurrence was evident in 30%. Radkowski stage and type of treatment did not differ significantly within the EOJNA group (*p *= 0.440 and *p *= 0.659, respectively).

**Conclusion:**

This systematic review suggests that rare cases of EOJNA have distinct disease characteristics. Patients in this cohort appeared to have more advanced disease and higher recurrence rates when compared with reported averages. We hope that this review prompts increased clinical awareness of this potentially more aggressive subtype of JNA. As more cases of EOJNA are reported, a more powered statistical analysis of this cohort would be feasible.

## Introduction

Juvenile nasopharyngeal angiofibroma (JNA) is a benign, locally aggressive fibrovascular tumor that arises primarily in the nasopharynx/posterior nasal cavity of adolescent males. It accounts for 0.05% to 0.5% of all tumors arising in the head and neck, with a reported incidence of 1 in 5000 to 1 in 60,000 in the US annually [2–5, 7]. It is the most common benign tumor arising in the nasopharynx of young males. Patients with JNA tumors frequently present with unilateral nasal obstruction and recurrent epistaxis [3]. Other presenting symptoms may include headache, facial swelling, anosmia, cranial neuropathy, otologic symptoms, and orbital abnormalities [1, 8].

JNA tumors are believed to arise adjacent to the sphenopalatine foramen. Recent reports have suggested a more specific site of origin, specifically, at the vidian canal orifice in the pterygopalatine fossa [9, 10], or possibly within the palatovaginal canal [11]. As the tumor grows, it may expand along various vectors of spread. Posteriorly, these tumors often invade along the vidian canal into the basisphenoid and pterygoid wedge. Laterally, they may spread into the pterygopalatine fossa which may cause displacement of the posterior wall of the maxillary sinus. This anterior bowing is a common computerized tomography (CT) radiological finding in patients with JNA tumors and is known as the Holmann Miller sign. From there, the tumor may continue to spread within the pterygopalatine fossa as well as laterally to the infratemporal fossa. Medial growth frequently deviates the nasal septum causing unilateral obstruction. Superiorly, the tumor may also spread and invade through the sphenoid sinus and cavernous sinus. Advanced stages of JNA are associated with intracranial invasion, which occurs in approximately 4–11% [2, 4]. Imaging is an important step in evaluating the extent of disease. In general, CT scans are ideal for evaluating the extent of bony invasion. Whereas, magnetic resonance imaging (MRI) is better at assessing the status of adjacent soft tissue structures such as the carotid artery [8] and intracranial invasion. Angiography confirms vascular supply and allows for the possibility of preoperative embolization if indicated [1]. Over ten different staging systems have been proposed, all of which are generally based on anatomic tumor extension. The Radkowski stage is the most frequently used staging system [12], however, other options like Chandler’s [13] stage or Andrew’s (modification of Fisch) [14] staging system may be applied. The University of Pittsburgh Medical Centre staging system for JNA was more recently created in 2010 and is geared more towards endoscopic resection and takes embolization status into account [15].

The definitive management of JNA tumors generally involves surgical resection. This may be performed via an endoscopic, external or a combination of these two surgical approaches [1]. Endoscopic excision has become more commonplace in the past two decades. This is in large part due to excellent visualization combined with reduced invasiveness with potentially diminished iatrogenic blood loss, and other associated complications [1, 4]. Open surgical approaches (e.g. lateral rhinotomy, infratemporal, transmaxillary, Le Fort I, with/without endoscopic assistance) are typically reserved for more advanced disease states that would present difficulty for complete endoscopic excision [1–3]. Radiotherapy is also occasionally employed, but is reserved for clinical scenarios where surgical excision would have high likelihood of associated morbidity, such as tumors with major intracranial or internal carotid extension [1]. Recurrence risk is significant and ranges between 13 and 46% and is largely related to tumor characteristics (e.g. size, location, extension) and possibly treatment modality [1, 16].

Onset of JNA is typically within the adolescent years, with an overall mean ranging between 13 and 22 years of age [1–6]. Liu et al. proposed that this preponderance for adolescent males may be related to increased expression of androgen receptors within these tumors, suggesting that their growth is driven hormonally [17]. Schick et al. have alternately proposed that incomplete regression of a branchial artery during embryogenesis leads to the formation of JNA [18]. Oncogenic mutations in C-MYC and C-KIT have also been described [19]. However, several authors have reported more extreme ranges for age of presentation. For example, Boghani et al. [3] report an age range of 1.25–64 years in a systematic review of 1047 JNA cases. In addition, Huang et al. report an age range of 8–41 years in a systematic review of 162 JNA cases [4] Pre-pubertal patients at the youngest extremes of these cohorts pose an interesting incongruence, as their androgen levels are much lower than their post-pubertal counterparts. Upon reviewing the literature, there is a paucity of studies looking specifically at cohorts of EOJNA, and in fact many series quote an age range of greater than 10 years old.

The primary purpose of this systematic review was to evaluate disease characteristics (e.g. tumor stage) and treatment outcomes in this rare subgroup. We also compared early-onset disease characteristics with data from all-age JNA cohorts.

## Case example

**Fig. 1:**
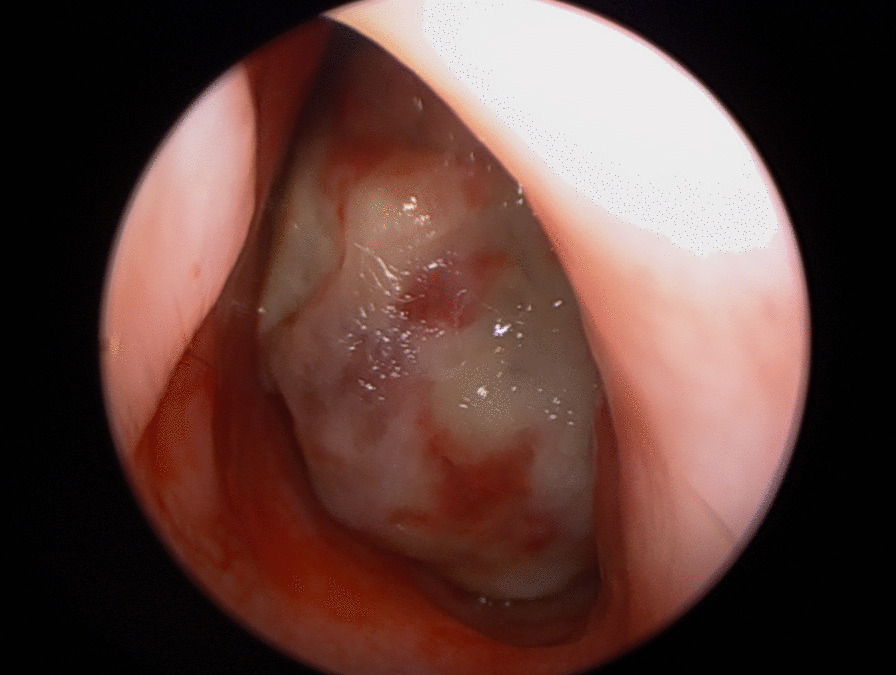
A photograph of an endoscopic evaluation of the left nasal passage revealing a large, pulsatile, grey mass

**Fig. 2:**
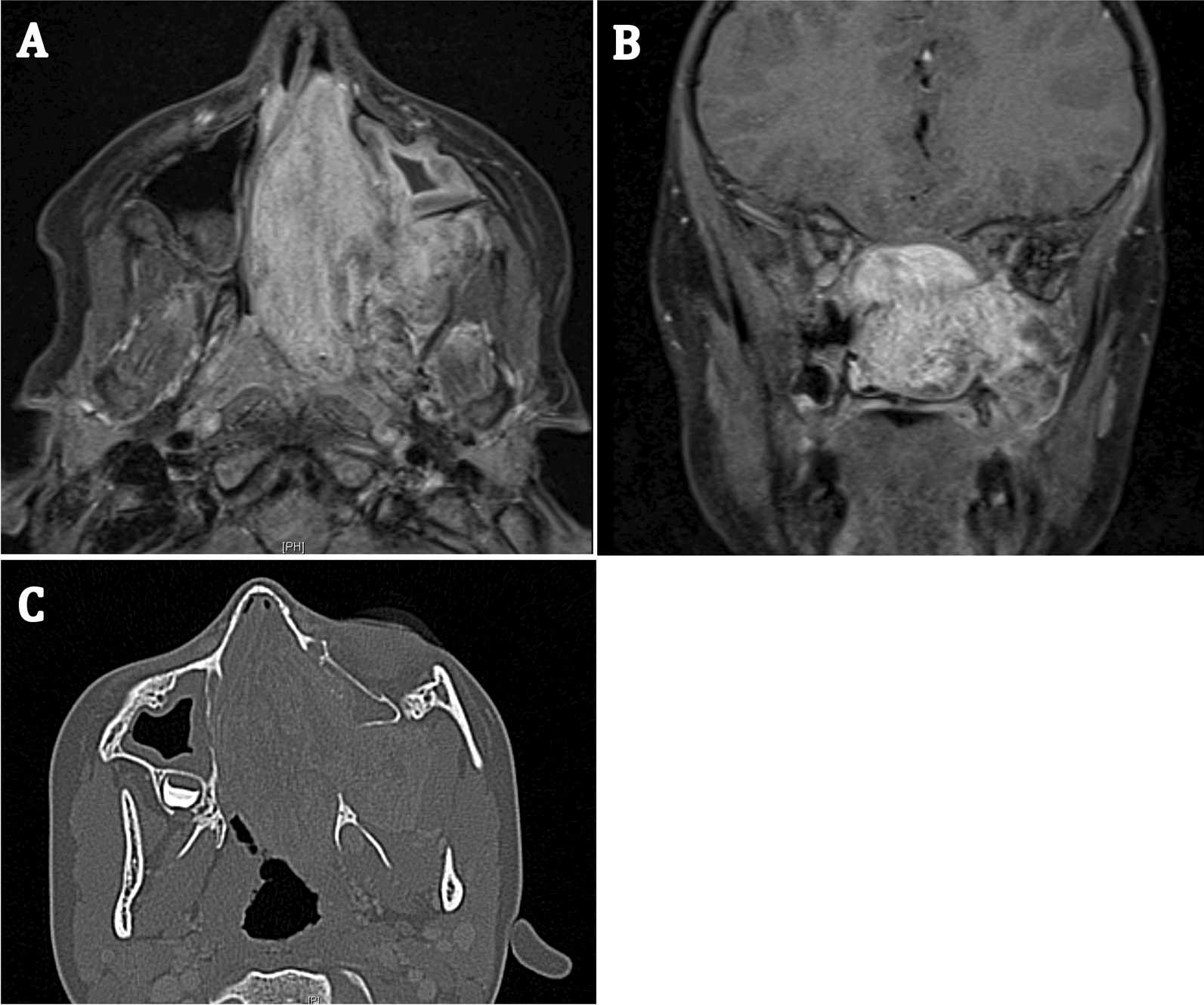
**A** Axial MRI view of the large JNA in the entire nasal cavity and the pterygopalatine fossa. **B** Coronal MRI view showing the same. **C** Axial CT view of the tumor

**Fig. 3:**
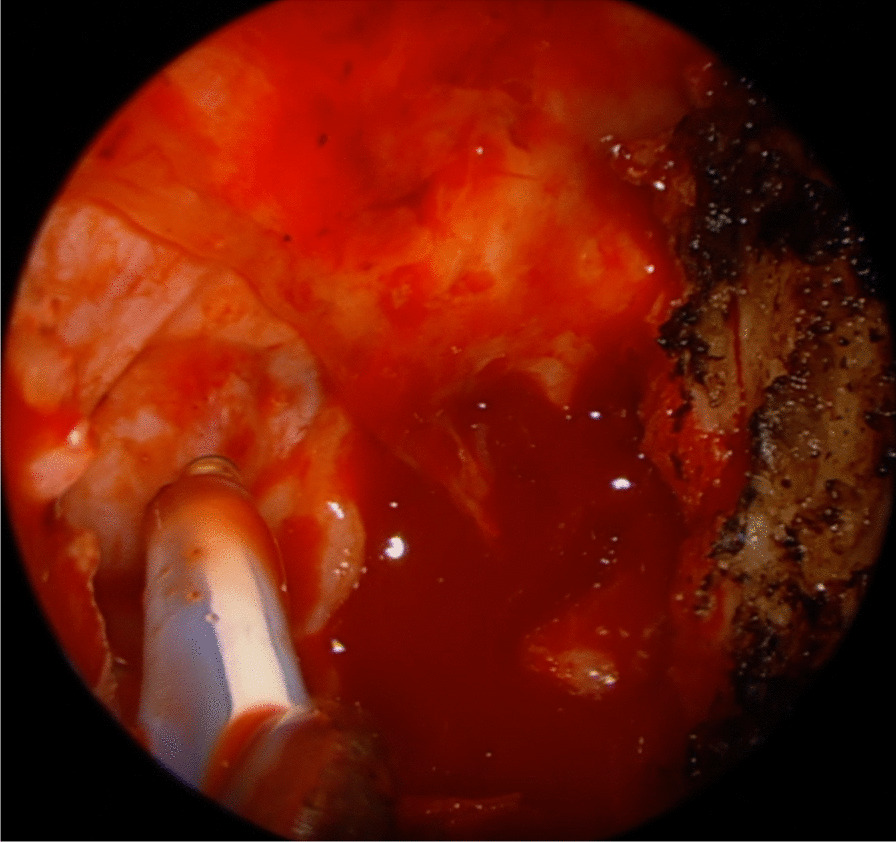
A clinical intraoperative photograph showing a dehiscent internal carotid artery in the cavernous portion. The Doppler probe is pointing at the artery with no bone covering

A 7-year-old boy presented with a one-year history of nasal obstruction and worsening epistaxis over a 6-month period. Intranasal endoscopic evaluation (Fig. 1) revealed a large pulsatile grey mass. CT and MRI evaluation were consistent with a JNA (Fig. 2). Preoperative embolization angiography revealed blood supply from the ipsilateral internal carotid (ICA), internal maxillary and ascending pharyngeal arteries. An endoscopic trans-pterygoid approach utilizing a modified Denker’s maxillectomy was successfully performed with removal of the tumor and 350 mL of blood loss. A microdoppler (Fig. 3) was utilized to identify bilaterally dehiscent ICA’s. Of note, although the vidian nerve was sacrificed, the greater palatine branch of V2 was preserved and retracted laterally to prevent palatal anesthesia. This patient was noted to have a recurrence in the pterygoid wedge approximately 2.5 years after the initial procedure. This was again addressed endoscopically and the patient is currently asymptomatic and being followed with serial MRI’s as well as clinically/endoscopically.

## Methods

### Literature search strategy and selection criteria

In accordance with Preferred Reporting Items for Systematic Reviews and Meta-Analyses Guidelines (PRISMA 2020), we performed a systematic review to investigate the EOJNA, defined by our group as an age of onset less than 10 years of age. We searched Embase, Cochrane database and MEDLINE from 1996 to February 2021 for studies that reported cases of JNA in this age group. These databases were searched using keywords including: “*nasopharyngeal angiofibroma”* OR *“nasal angiofibroma”* OR “*juvenile angiofibroma”*. Articles were screened and assessed for eligibility independently by 2 review authors (M.N and T.M). We included case reports, case series, cohort studies, and systematic reviews. In accordance with our study objective, only EOJNA cases were included. If possible, for studies presenting data with a variety of different age groups, only data pertaining to those identified as EOJNA were extracted.

### Data synthesis and statistical analysis

Study characteristics and relevant patient data were recorded. This included age, gender, presenting symptoms, Radkowski stage, primary treatment modality, and recurrence status. A Chi-Square test was used to assess for any significant differences in recurrences rate when patients were stratified by Radkowski stage or treatment modality. Reported averages for JNA in all ages were compiled from three studies to use as a control group for comparison [3, 4, 16, 20]. Data was compiled in Microsoft Excel Version 15.38. Statistical analysis was done using IBM SPSS Version 20. Statistical significance was defined as a p-value of less than 0.05.

## Results

### Description of studies and patient demographics

**Fig. 4:**
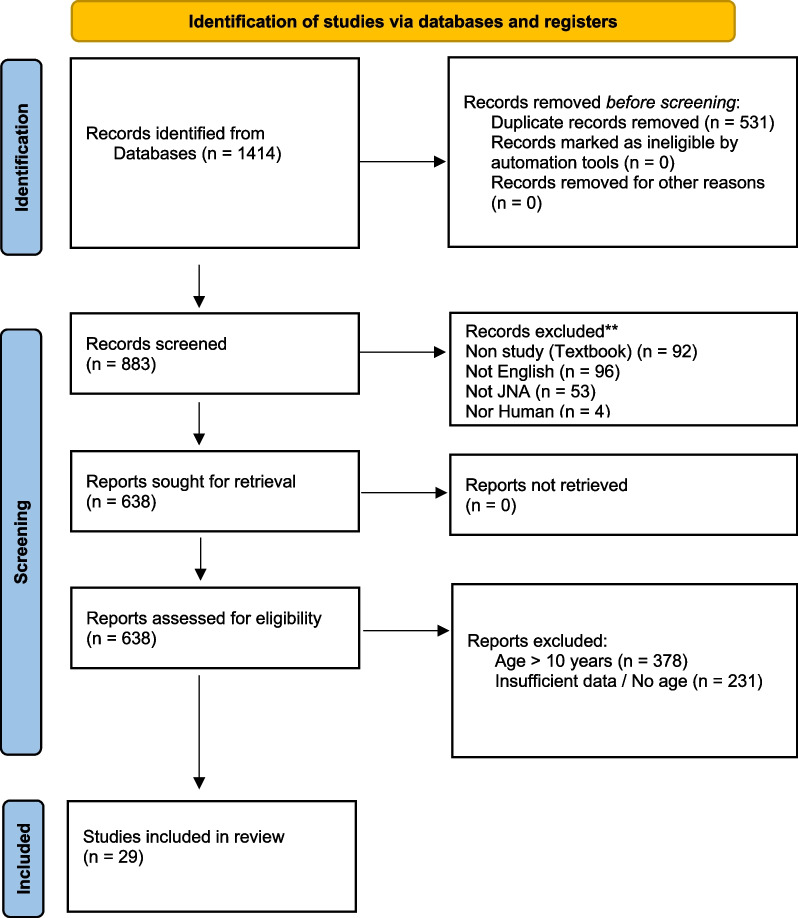
Flowchart detailing search strategy and reasons for exclusion

The initial database search yielded 1414 results, and 883 studies remained after duplicates were removed. All 883 studies were then independently reviewed with exclusions based on abstract review. If any uncertainty remained regarding study eligibility after abstract review, the full text was reviewed. Studies were excluded for several reasons, the most common of which was that study patients were ≥ 10 years of age (n = 378). Other reasons for exclusion included: insufficient data (n = 231), textbook chapter (n = 92), not English (n = 96), not JNA (n = 53), and not human (n = 4). “Insufficient data” typically referred to studies that did not provide the age of included patients or that did not provide specific data for patients less than 10 years of age. At the conclusion of this process, 29 studies met our inclusion criteria [1, 2, 4–8, 21–46]. Figure 4 illustrates the details related to our study selection process.

A total of 34 JNA cases under age 10 at presentation were identified in the literature. Of these, 17 (50.0%) were 9 years old at presentation, 12 (35.3%) age 8, 4 (11.8%) were 7, and 2 cases aged 6 and 4. The average age was 8.15 years (SD = 1.08). Study designs included retrospective cohort studies (n = 12), case series (n = 10), case reports (n = 6), and 1 was a prospective cohort study. Patient follow-up time was reported in 19 of the included studies with an average of 32 months (SD = 41.42). There were five instances in which studies contributed multiple JNA patients under 10 years of age; [2, 23, 27, 29, 44]. The gender breakdown was overwhelmingly male in this cohort with 31 (91.2%) male cases and 3 (8.8%) female cases. Table 1 summarizes the study characteristics and patient demographics of the included studies.

**Table 1:** Characteristics of included studies

Study	Study design	No. of patients	No. of Patients < 10 years	Age (years)	Sex	Follow-up
Gaillard [40]	R	16	1	9	M	7 yrs
Kalani [8]	R	22	1	9	M	1 mo
Ardehali [5]	R	47	1	7	M	2.5 yrs
Ferreira [39]	P	9	1	9	M	1 yr
Handa [22]	C	1	1	8	M	6 mo
Baptista [38]	C	1	1	8	F	6 mo
Salcone [37]	C	1	1	9	M	6 mo
Gupta [23]	CS	8	2	1: 4 2: 6	1: M 2: M	1: 1.5 yrs 2: 1 yrs
Garofalo [2]	R	12	2	1: 9 2: 9	1: M 2: M	1: 15 yrs 2: 8 yrs
Bakshi and Bhattacharjee [47]	C	1	1	9	M	5 mo
Moorthy [21]	R	13	1	7	M	3 yrs
Yi [6]	R	51	1	8	M	1 yrs
Cruz [4]	R	19	1	9	M	–
Szyfter [25]	R	15	1	9	M	–
Punj [36]	R	56	1	8	M	–
Lv [35]	R	22	1	9	F	–
Lutz [34]	R	15	1	9	M	–
Yamada [33]	CS	11	1	8	M	–
Cansiz [32]	CS	22	1	9	M	–
Yamada [33]	C	1	1	8	M	1.25 yrs
El-Banhawy [26]	CS	20	1	8	M	6 yrs
Fonseca [31]	CS	15	1	8	M	3 yrs
Browne [29]	CS	5	2	1. 8 2. 9	1. M 2. M	1. 4 yrs 2. 11 yrs
Lee [27]	R	27	2	1. 8 2. 9	1. M 2. M	–
Dubey [28]	CS	16	1	7	M	–
Tseng [30]	C	1	1	9	M	6 mo
Gruber [44]	CS	2	2	8 7	M F	2 yrs 3 yrs
Pletcher [45]	CS	23	1	8	M	–
Donald [46]	CS	5	1	9	M	1 mo
Average (SD)				8.15 (1.08)		32.0 mo. (41.42)

*R* Retrospective cohort study, *P* Prospective cohort study, *C* Case report, *CS* Case series

### Presenting symptoms and stage at presentation

Several presenting symptoms commonly associated with JNA growth were identified in this cohort. Symptoms were reported in 23 of 34 patients. The remaining 11 patients did not have data available regarding presenting symptoms. Patients most commonly presented with nasal obstruction (n = 15, 65.2%) and epistaxis (n = 14, 60.9%). Other associations included proptosis (n = 5, 21.7%), sleep disordered breathing (n = 4, 17.4%), and visual changes (n = 4, 17.4%). Ocular manifestations included decreased visual acuity, strabismus, and diplopia. Headache (n = 3, 13.0%) and facial swelling (n = 2, 8.7%) were also noted. Other nasal symptoms such as hyposmia (n = 1, 4.3%) and hyponasal speech (n = 1, 4.3%) were rarely reported in this cohort. An outline of presenting symptoms is provided in Table 2.

**Table 2:** Overview of presenting symptoms

Presenting symptoms (n = 23)	No. reported	% reported
Nasal obstruction	15	65.2
Epistaxis	14	60.9
Proptosis	5	21.7
Sleep disordered breathing	4	17.4
Visual changes	4	17.4
Headache	3	13.0
Facial swelling	2	8.7
Hyponasal speech	1	4.3
Hyposmia	1	4.3
Difficulty breathing	1	4.3

Overall stage at presentation was reported in 33 of 34 patients. As was previously discussed, the multitude of different staging systems creates a significant source of heterogeneity. The Radkowski and Fisch systems were most commonly utilized, in 6 and 4 cases respectively. Other staging scores used include Onerci, Andrews, Chandler, and Sessions staging. To minimize heterogeneity, primary staging for each patient was converted to a Radkowski stage using the original staging system presented or descriptions of tumor location and extension obtained from imaging or intraoperative accounts. Twelve patients (36.4%) were stage II at diagnosis, while 14 patients (42.4%) were stage III at diagnosis. Only 7 patients (21.2%) were stage I at diagnosis. The youngest patients, aged 6 and 4, were classified as stage III. Of patients that were 9 years of age, which was the most prevalent age in this cohort, 7 (43.8%) were stage II while 6 (37.5%) were stage III and 3 (18.8%) were stage I. An overview of stage at presentation vs. age is provided in Table 3.

**Table 3:** Stage at presentation vs. Age

		Radkowski stage (n = 33)			Total
		Stage I	Stage II	Stage III	
Age (years)	4			1	1
	6			1	1
	7		4		4
	8	4	1	6	11
	9	3	7	6	16
	Total	7 (21.2%)	12 (36.4%)	14 (42.4%)	33

### Treatment modalities, complications, and recurrence

Primary treatments reported in this cohort included surgery, radiation, or a combination of the two. This was outlined in 33 of 34 cases. Surgery was the most common primary treatment modality employed (n = 30, 90.9%). External surgical approaches such as lateral rhinotomy and transmaxillary approaches were cited most commonly in the surgical group at 66.7% (n = 22). In 3 external surgical cases, endoscopic assistance was utilized. A primary endoscopic approach was used in 24.2% (n = 8) of cases. Of note, 24.2% (n = 8) of cases managed with surgical excision required multiple operations, most commonly transitioning from an initial endoscopic approach to open approach. Postoperative radiation was given to 1 patient. Radiotherapy alone was used as a primary treatment modality in only 9.1% (n = 3) of cases. All three of these cases had intracranial and orbital involvement.

The most common complication reported in this cohort was excessive intra-operative blood loss (n = 9), which was defined as > 500mL. This occurred in 27.3% (n = 6) of patients that underwent primary open surgical excision and in 37.5% (n = 3) of patients that underwent primary endoscopic surgical excision. Blood transfusion was required for 13.6% (n = 3) of patients that had open surgery and 12.5% (n = 1) of patients that had endoscopic surgery. All patients that required blood transfusion lost ≥ 1L intra-operatively. Other complications reported in the non-endoscopic surgical group included post-operative trismus (9.1%, n = 2), cavernous sinus injury (9.1%, n = 2), lateral rectus paralysis (4.5%, n = 1), and hyperlacrimation (4.5%, n = 1). No additional complications were reported for patients that underwent endoscopic surgical excision. Cataracts were reported as a complication in 100% of patients (n = 3) that underwent primary radiotherapy. Growth retardation was reported as a complication following radiotherapy in 1 subject. An overview of reported complications and their relation to treatment modality is outlined in Table 4.

**Table 4:** Blood loss and other complications vs. treatment modality

Treatment (n = 33)	No. reported	Mean blood loss (mL)	No. requiring transfusion	Other reported complications
Open surgery	22	1606 (n = 14)	3	Post-op trismus (n = 2) Cavernous sinus injury (n = 2) Lateral rectus paralysis (n = 1) Hyperlacrimation (n = 1)
Endoscopic surgery	8	675 (n = 4)	1	–
Radiation	3	–	–	Cataracts (n = 3) Growth Retardation (n = 1)

Table 5 illustrates the distribution of treatment modalities by Radkowski staging. Data regarding both treatment and staging were provided for 31 of 34 patients. Patients with stage I disease (n = 7) were managed with open surgery in 57.4% (n = 4) of cases and endoscopic surgery in 42.6% (n = 3) of cases. Of patients with stage II disease (n = 10), 70.0% (n = 7) were managed with open surgery, 30.0% (n = 3) with endoscopic surgery. Of patients with stage III disease (n = 14), 64.3% (n = 9) were managed with open surgery, 14.3% (n = 2) with endoscopic surgery, and 21.4% (n = 3) with radiotherapy. Follow up and assessment for tumor recurrence was reported in 28 of 34 patients. Average follow-up time was 32 months (SD = 41.4%). Recurrence was not reported for radiotherapy because this is not a curative treatment. Recurrence of tumor was identified in 28.6% (n = 9) of patients. Of those, 37.5% (n = 3) were following an endoscopic surgical approach and 25.0% (n = 5) were following an open surgical approach. The difference in recurrence rates between open surgery and endoscopic surgery was not statistically significant (*p *= 0.334, ^2^ = 0.933). In addition, recurrence risk was also analyzed in association with the primary Radkowski stage. 33.4% (n = 7) of stage II and III patients recurred, whereas only 14.3% (n = 1) of stage I patients recurred. However, this difference did not reach statistical significance (*p *= 0.595, ^2^ = 1.037).

**Table 5:** – Treatment modality and recurrence rate by Radkowski stage at presentation

	Radkowski stage (n = 29)			Total
	Stage I	Stage II	Stage III	
Open surgery	4	7	9	20
Recurrence	1 (25.0%)	2 (28.6%)	2 (22.2%)	5 (25.0%)
Endoscopic surgery	3	3	2	8
Recurrence	0	1 (33.3%)	2 (100%)	3 (37.5%)
Total recurrence	1 (14.3%)	3 (30.0%)	4 (36.4%)	
Radiotherapy	0	0	3	3
OS versus ES	*p *= 0.334 (χ^2^ = 0.933)			
Stage I versus II versus III	*p *= 0.595 (χ^2^ = 1.037)			

A Chi Squared test was used to compare the recurrence rate between open surgery (OS) and endoscopic surgery (ES). The same was also used to compare recurrence rate between Radkowski stage I, II, and III

## Discussion

Juvenile nasopharyngeal angiofibroma is reported to occur primarily in adolescent males, likely related to large numbers of androgen receptors within these tumors [17]. However, there are large cohorts in the literature that report considerable range with regards to age of onset. Of particular interest to this review were cases of EOJNA, presenting in children < 10 years of age. A hypothesis regarding etiology of JNA involves boys reaching puberty. There are many different studies which generally state that on average boys start puberty between ages of 9 and 14 (variable depending on source) [48]. The development of JNA before a male reaches puberty potentially differentiates itself from the typical JNA patient. 10 years was chosen as early-onset since the vast majority of boys have yet to reach puberty at this age.

The progression of this disease in pre-pubertal androgen environments, especially in the two youngest patients (age 4 and 6), suggests that other mechanisms of pathogenesis are playing a significant role. There is also the possibility that precocious puberty (PP) has driven tumor growth in these patients. PP is defined as development of secondary sexual characteristics before age 8 in females and age 9 in males. The incidence of PP has been consistently rising in the past decades, with current estimates ranging between 1 in 5000–100,000. Recent studies have suggested that the majority of PP is idiopathic, with only 26–40% of PP cases arising from organic causes such as central nervous system tumors [49]. In addition, there were a relatively large proportion of females within this group at 8.8%. In contrast, only 0.7% of patients were female when reviewing the literature [3, 4, 16, 20]. This may also call into question the essential role of androgens in the development of EOJNA, or perhaps there is an exogenous source of androgens common to both male and female patients in this cohort.

Only a total of 34 EOJNA cases (35 including our case) were identified in our review, which eludes to the fact this presentation is very rare and makes this a difficult entity to study. Nevertheless, disease severity appears to be more extensive in patients with EOJNA when compared with all-age cohorts reported in the literature [3, 4, 16, 20]. Within the early-onset cohort, 57.6% of patients presented as stage I or II, while the reported average in the literature is 86.4%. Conversely, 42.4% of EOJNA patients had advanced stage III disease, while only 13.6% of patients were stage III in the literature. There seems to be a disproportionate number of EOJNA patients presenting with advanced (stage III) disease. It is possible that this represents a more aggressive, early-onset clinical subtype. However, there is also the possibility that due to decreased body awareness and communication skills, symptoms in this age group are not identified until disease has progressed further. Furthermore, early diagnosis of JNA may be missed as other etiologies of nasal obstruction and epistaxis are favored in the context of low clinical suspicion. Many otolaryngologists do not routinely scope for JNA in children less than 10 years of age.

Overall, a higher proportion of patients with EOJNA were treated with open surgery when compared with reported averages for adolescent JNA patients. Within the early-onset cohort, 66.7% of patients were treated with open surgery while only 24.2% were treated endoscopically. On average 51.6% of patients had open surgery while 48.1% had endoscopic surgery upon review of available literature. This may be a related to the larger proportion of patients with advanced disease in this cohort, or perhaps the belief amongst some surgeons that the smaller nostrils and nasal cavities of these patients would present too difficult a challenge for endoscopic surgery. In addition, the rate of tumor recurrence was higher in the early-onset cohort. Two recent meta-analyses of JNA surgical treatment suggest that, in general, the endoscopic approach results in a lower likelihood of recurrence [50]. Of early-onset patients, 28.6% had at least one reported recurrence, while the reported average was 20.7%. This seems to suggest higher recurrence rates and worse outcomes for EOJNA patients. This is further supported by Rowan et al. [51], who found that the average age of JNA patients that required treatment for residual disease was significantly lower than patients that had stable residual disease.

Presenting symptoms in EOJNA patients appear similar to typical adolescent JNA symptoms described in the literature [3, 4, 16, 20]. Nasal obstruction and epistaxis were the two most common presenting symptoms in both groups, with very similar incidence figures. Nasal obstruction was present in 87.3% patients when reviewing the literature and in 65.2% of the early-onset cohort. Similarly, the reported average for epistaxis was 86.5%, while 60.9% of patients in the early-onset cohort presented with epistaxis. Interestingly, proptosis and sleep disordered breathing were much more prevalent in the early-onset cohort. Proptosis was reported in 21.7% of the early-onset cohort while on average 12.9% presented with this in the literature. Likewise, sleep disordered breathing was reported in 17.4% of the early-onset cohort but was present in only 3.1% upon literature review. This could be related to the higher proportion of advanced disease in the early-onset cohort, potentially compounded by the fact that patients in this age group may still have significant adenotonsillar hypertrophy. The prevalence of headache, facial swelling, hyposmia and visual changes were similar between the two groups. Also of note, hyponasal speech was reported much less frequently in the early-onset cohort (4.3%) compared to averages in the literature (41.3%).

## Limitations

### Sample size

This study presents valuable insights into patients with EOJNA, however, findings are significantly limited by our small sample size. With only 34 total cases of EOJNA identified in the literature, we have established that this is a very rare occurrence. The ability to appropriately analyze our data and assess significant relationships was restricted. In particular, our non-significant Chi Squared test results were likely impacted by our low power cohort size.

### Study population

Selection bias and sampling bias are likely contributing to some degree in our findings. Although attempts to reduce bias associated with study selection were made with a standardized search protocol, Embase, Cochrane database, and MEDLINE may provide somewhat limited access to cases published outside of North America and Europe. Included studies consisted of case reports, case series, and cohort studies, which lacked systematic selection criteria for its patients. It is likely that cases presented in the literature were of more advanced staging, or required more extensive surgical management than what is actually true for this population. As well, a significant number of cohort studies were specifically looking at endoscopic surgical management for JNA, potentially inflating our observed rate for this treatment modality.

### Reporting measures

There was considerable diversity of reporting measures used in the included studies. Although disease stage for patients was converted to the Radkowski system, there were 5 other staging systems encountered. This may bias our results, as there is the potential for ceiling or floor effects depending on the scale. Standardized reporting of surgical approach and intraoperative complications was also lacking in this study, especially when patients underwent multiple surgeries. Although the average follow-up time reported was 32 months, this data was only included in 19 of the included patients and follow-up was as short as 1 month in one study. As a result, attrition bias could be affecting our findings, specifically those relating to disease recurrence. Furthermore, in most cases the time to recurrence after surgery was not reported. This information could allow for more thorough analysis of recurrence with respect to treatment modality and stage.

### Control group

Although we compared our results with reported averages in the literature, this was not a true control group. The data we made comparisons with were from studies including JNA patients of all ages. Ideally a more appropriate control group would be comprised exclusively of patients greater than ten years of age, and should be recruited prospectively. This could create a more internally valid statistical analysis of these two cohorts.

## Conclusion

In conclusion, this systematic review identified only 34 cases of JNA in children under 10 years of age, indicating that development of disease within this age group is rare. We found disease characteristics unique to this cohort when compared with JNA patients of any age. Disease progression tended to be more extensive, with a higher rate of open surgical excision when compared with reported averages. Furthermore, disease recurrence appeared to be more common in EOJNA. We anticipate that this review prompts increased clinical awareness of JNA manifesting in young patients, and that this may represent a more aggressive clinical subtype. As more cases are presented in the literature, an examination with a more well defined control group (> 10 years of age) and a more powerful sample size would advance our understanding of this condition. As well, more extensive research may further elucidate the role that androgens, and other growth factors such as VEG-F, play in the growth of JNA and more specifically EOJNA. Consideration of precocious puberty and exogenous androgens would also be a valuable avenue for further research.

## Data Availability

The datasets used and/or analyzed during the current study are available from the corresponding author on reasonable request. All data generated or analyzed during this study are included in this published article under methods.

## Abbreviations

JNA: Juvenile nasopharyngeal angiofibroma
EOJNA: Early-onset JNA
CT: Computerized tomography
MRI: Magnetic resonance imaging
ICA: Internal carotid artery
PP: Precocious puberty
